# Exercise and Oxidative Stress Biomarkers among Adult with Cancer: A Systematic Review

**DOI:** 10.1155/2022/2097318

**Published:** 2022-02-18

**Authors:** Yari Longobucco, Alice Masini, Sofia Marini, Giuseppe Barone, Carmela Fimognari, Laura Bragonzoni, Laura Dallolio, Francesca Maffei

**Affiliations:** ^1^Department of Medicine and Surgery, University of Parma, Parma, Italy; ^2^Department of Biomedical and Neuromotor Science, University of Bologna, Bologna, Italy; ^3^Department for Life Quality Studies, University of Bologna, Campus Rimini, Italy

## Abstract

Evidence shows that exercise can have a favourable effect in cancer patients. The exercise's clinical benefits are likely to concern multiple interrelated biological pathways, among which oxidative stress plays a key role. Regular training can induce an adaptive response that strengthens the antioxidative status of the body. To formulate public health recommendations regarding the optimal exercise prescription for cancer patients, a detailed understanding is needed regarding the effect of exercise on variables linked to oxidative stress and antioxidant status of patients. The goal of this systematic review, based on PRISMA, was to explore and critically analyse the evidence regarding the efficacy of exercise on oxidative stress biomarkers among people with cancer. Study search was conducted in the following databases: PubMed, Cochrane, CINAHL, Embase, PEDro, and SPORTDiscus. The studies' quality was assessed with the Cochrane risk-of-bias tool and STROBE scale. After identification and screening steps, 10 articles were included. The findings provide an encouraging picture of exercise, including resistance training and aerobic activities, in people with cancer. The exercise improved the indicators of the total antioxidant capacity, increased the antioxidant enzymes' activity, or reduced the biomarkers of oxidative damage in various forms of cancer such as breast, lung, head, and neck. Regarding oxidative DNA damage, the role of exercise intervention has been difficult to assess. The heterogeneity of study design and the plethora of biomarkers measured hampered the comparison of the articles. This limited the possibility of establishing a comprehensive conclusion on the sensitivity of biomarkers to estimate the exercise's benefits. Further high-quality studies are required to provide data regarding oxidative stress biomarkers responding to exercise. This information will be useful to assess the efficacy of exercise in people with cancer and support the appropriate prescription of exercise in anticancer strategy.

## 1. Introduction

Exercise training is, in general, secure for people that survived after cancer diagnosis, and each of them should remain physically active, as stated in the report of 2018 of the American College of Sport Medicine entitled: “Internationally Multidisciplinary Roundtable on Physical Activity and Cancer Prevention and Control.” It should be clarified that physical activity (PA) is defined as any movement of the body generated by skeletal muscles that required energy expenditure. PA can be classified into sports, occupational, conditioning, household, or other activities [1]. Likewise, exercise is a subcategory of PA that is planned and structured, directed also at improving or maintaining cardiofitness status [2].

There is evidence showing that exercise has positive effects in patients diagnosed with cancer [3, 4]. Adapted exercise interventions can diminish the possible resurgence of tumour growth in breast, colon, and prostate cancer [5, 6]. Furthermore, exercise is associated with a better survival and an attenuation of the negative consequences of chemotherapy and radiation [5, 7, 8].

In people with cancer, exercise is linked to positive modifications in cardiorespiratory fitness, physical function, and in anthropometric composition, as well as in patient-reported health benefit in quality of life and manage of fatigue [9–11]. Recent findings indicated that exercise could play a key role in tumour biology. Evidence suggests that exercise could downregulate a group of RAS family oncogenes (RAN, RAB14, and RAB8A) [6–12]. Moreover, the capacity of exercise to enhance the endogenous antioxidant defences has been postulated as a contributing factor to counteract the oxidative stress in various phases of tumourigenesis [6–13]. It is well known that oxidative stress-induced DNA damage can promote the development and progression of cancer. Moreover, cancer cells themselves increase the levels of reactive oxygen species (ROS), inducing cancer progression [14, 15]. In this framework, systematic exercise training can improve physical fitness and capacity of the patients through enhancing the antioxidative status of the body. Although acute and exhaustive training increases in ROS production, the moderate exercise (chronic exercise or aerobic training) induces an organism's response with a decrease in ROS generation and an improvement in antioxidant status [6, 13, 16]. Besides, regular exercise has a hormetic effect given that low levels of oxidative stress deriving from cells as a consequence of exercises may trigger cellular mechanisms which promote the tolerance of acute oxidative stress [6–17]. At a molecular level, exercise training stimulates a transient production of ROS, and it can activate a redox circuit linked [18]. More specifically, ROS arose during exercise and activated the transcription of nuclear factor erythroid 2 (Nrf2). Nrf2 plays a fundamental role in the *trans*-activation of the antioxidant response element (ARE) and in the upregulation of various proteins involved in antioxidant defences [18, 19]; thus, it is pivotal in preserving the balance in the cellular redox process. Most of the Nrf2-dependent target genes encode for enzymes that protect DNA, proteins, and lipids from ioxidative damage. These genes take part in the synthesis of antioxidant enzymes such as catalase (CAT) or superoxide dismutase (SOD), glutathione and other stress response [20–22].

On this basis, physical exercise can be regarded as a regulator of cellular redox homeostasis, which induces an adaptation to overcome the oxidative stress. However, the potential benefits of exercise are influenced by intensity, type, and duration of training [16].

In order to adopt public health recommendations on the optimal exercise prescription for adults with cancer, a more detailed understanding is needed regarding the effect of exercise on variables linked to oxidative stress and antioxidant status. Usually, changes in homeostasis redox are measured by analysing different biomarkers, mainly in the blood or in urine samples collected in test populations. Such biomarkers can detect a specific type of damage on lipids, proteins, and DNA or the concentration of enzymatic and nonenzymatic antioxidants. Thus, different biomarkers can be evaluated for any molecular or cellular damage that can be caused by ROS [23].

In this context, the primary aim of this article is to explore and investigate the evidence regarding the exercise's effect on oxidative stress biomarkers among postdiagnosis cancer patients. Furthermore, a detailed evaluation of previous research is also addressed regarding the association by type, dose, and timing of exercise and cancer location.

## 2. Methods

### 2.1. Search Strategy

This systematic review was conducted according to PRISMA [24]. We registered the protocol of the systematic review in the International Prospective Register of Systematic Reviews (ID: CRD42021258326). The primary research objective was addressed, through the development of the PICO question (Patients, Interventions, Comparators, and Outcomes) using the following search terms: (P) people with cancer diagnosis, (I) physical activity exercise intervention, (C) usual treatment and/or no exercise intervention, and (O) the efficacy of exercise on oxidative stress biomarkers.

We conducted a systematic literature search in PubMed, Cochrane Library, CINAHL, Embase, PEDro, and SPORTDiscus up to May 2021 to screen all articles focused on the effect of structured exercises treatment on oxidative stress biomarkers in people with a diagnosis of cancer.

The electronic databases were searched, with a publication date limit of 10 years, because we were focused on recent treatments and approaches. Specific criteria were applied in the search approach: we included randomized controlled trials (RCTs), quasiexperimental study, clinical study, clinical trial, case report, and observational study, with full text available. Search terms was created using the following keywords and Boolean terms: ((Post diagnosis Cancer OR Neoplasia OR Neoplasm OR Tumo^∗^ OR Cance^∗^ OR Malignan^∗^ OR Malignant Neoplas^∗^ OR Neoplas^∗^) AND (Exercis^∗^ OR Physical Activit^∗^ OR Activities Physical OR Activity Physical OR Exercise Physical OR Exercises Physical OR Physical Exercise OR Physical Exercises OR Acute Exercis^∗^ OR Exercise Acute OR Exercises Acute OR Exercise Aerobic OR Aerobic Exercis^∗^ OR Exercises Aerobic OR Exercise Training OR Exercise Trainings OR Training Exercise OR Trainings Exercise OR Remedial Exercise OR Exercise, Remedial OR Exercises, Remedial OR Remedial Exercises OR Therapy Exercise OR Exercise Therapies OR Therapies, Exercise OR Rehabilitation Exercise OR Exercise, Rehabilitation OR Exercises, Rehabilitation OR Rehabilitation Exercises) AND (Oxidative Stresses biomarkers OR Stress Oxidative markers OR Antioxidative Stress OR Antioxidative Stresses OR Antioxidant enzymes OR Stress, Antioxidative OR Anti-oxidative Stress OR Anti oxidative Stress OR Anti-oxidative Stresses OR Stress, Anti-oxidative OR Antioxidant plasma status OR 8-hydroxy-deoxyguanosine)). When necessary, the search string was adapted to perfectly fit in each database.

A grey literature search was conducted using Medrxiv, and hand searches of key conference proceedings, journals, professional organizations' websites, and guideline clearing houses. Finally, using the snowball technique, we reviewed the primary and most important papers' references in order to find possible more studies.

### 2.2. Inclusion and Exclusion Criteria

The inclusion criteria were the following: (1) language: articles written in English; (2) study design: randomized controlled trial, quasiexperimental study, clinical trial, clinical study, case report, and observational study with original primary data; (3) population of interest: people with cancer diagnosis; (4) exercise experience: any type of exercise; (5) outcome measurement: oxidative stress biomarkers evaluation, oxidative biomarkers assessed at least once in the paper; additional physical performance measured outcomes, or other indices of physical performance described in each study for example, balance, mobility measured at least once in the study; and (6) comparison: standard treatment and/or no intervention.

The exclusion criteria were as follows: (1) articles not relevant for the research area, (2) people without cancer diagnosis, (3) absence of exercise intervention, and (4) research studies or other papers with no original data.  Table 1 summarized the PICOST eligibility criteria.

### 2.3. Data Extraction and Quality Assessment

The reviewers examined all the papers primarily by reading the titles and abstracts; then, the eligible articles were selected based on our PICOST. All potentially eligible studies were retrieved, after the removal of duplicates, extracted and then reviewed independently by the five researchers (LD, AM, SM, GB, and YL) using a scheduled data extraction format. Disagreements regarding the eligibility of the studies were resolved by discussion among all the authors. The data from the included studies were extracted by the researchers, following the standardized rules for studies collection. The details collected comprised: first's author's name, year of publication, country, study design, study population with ages and number of experimental (EG) and control (CG) groups, type intensity and frequency of the intervention, outcomes, and results. Results were described as mean ± SD where possible. The data extraction was based on the methods provided by the *Cochrane Reviewers' Handbook* [25]. Possible divergences were solved by consensus (LB, FM).

We contacted the study's authors when additional information was necessary [26].

The selected studies were assessed for the risk of bias separately by four researchers (LD, AM, SM, and GB), using the “Cochrane risk-of-bias tool for randomized trials” (ROB) [27] and the “STROBE statement checklists for observational studies” [28]. Any reviewers' disagreement, upon the quality scores, was solved in a schedule meeting manage by a fifth blind reviewer (YL). The risk of bias evaluation was made based on the oxidative stress biomarker outcomes. This methodological approach was endorsed by the PRISMA [24].

The Cochrane risk-of-bias tool for RCTs presents seven categories of bias: (1) how the randomization sequence was generated, (2) the allocation procedures' blindness, (3) selective reporting for reporting bias, (4) blinding of participants and personal, (5) the outcome evaluation procedures' blindness, (6) outcome data partially not reported, and “other bias” category, and (7) evaluated on the possible bias not reported in the previous domains. These categories are translated in a high, low, or unclear (when the authors did not provide enough information about the bias category) value of risk of bias. The STROBE scale is composed by 22 items divided into three different checklists: cohort study, cross-sectional, and case report studies [28]. In line with previous studies [29, 30], we adopted a cut-off for three scores: 0-14 poor quality, 15-25 intermediate qualities, and 26-33 good quality [31].

## 3. Results

### 3.1. Study Selection and Characteristics

**Figure 1:**
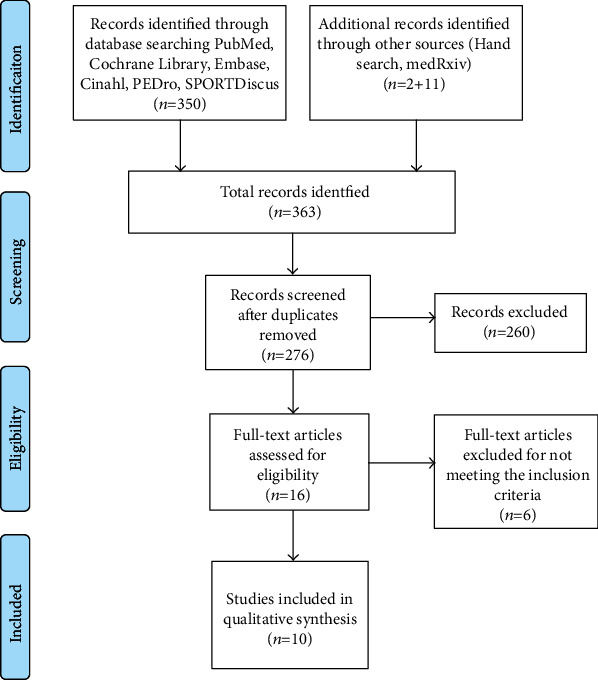
PRISMA flow diagram.

Overall, 363 articles were detected through the chosen databases and the hand search technique (Figure 1). Studies were published from 2011 to 2021; 87 were duplicated, and 260 studies were excluded in the first step of title and abstract reading. Finally, we considered 16 records as pertinent, 6 of which were subsequently excluded after full-text reading. The principal reasons for exclusion were linked to the nonmatch our review's aim: the effects of exercise interventions on oxidative stress biomarkers in people with cancer diagnosis. The prevalent reason of exclusion was due by the mismatch of the adopted inclusion criteria (people with cancer diagnosis). As shown in Figure 1, only 10 papers were included in the results.

### 3.2. Risk of Bias Assessment

On the basis of the descriptive analysis, we assessed the risk of bias. Experimental studies were analysed in accordance with the ROB tool for RCTs (Table 2). A RCT [32] showed an overall “good quality,” meeting all seven criteria of low risk of bias, the remaining four were evaluated as “poor quality.” Regarding items #1 and #2, Katsourakis et al. [33] described in detail how they obtained the random sequence and the allocation process, while this process was unclear for Karimi and Roshan [34] and at high risk of bias for both the studies of Repka and Hayward [35, 36], who adopted a pseudorandomization. All the studies showed a consistency between expected and reported outcomes, resulting in an evaluation of low risk of bias (item #3). Except for Jiang et al. [32], it was unclear the presence of other possible bias (item #4). There was no blinding of participants (item #5), but the researcher judged that the outcome is not likely to be affected by lack of blinding. Considering the blinding of outcome assessment (item #6), Jiang et al. [32] and Karimi and Roshan [34] described and applied techniques and methods that ensured the blindness; this aspect was unclear in both studies of Repka and Hayward [35, 36], while Katsourakis et al. [33] have been judged at high risk of bias. Finally, Jiang et al. [32] and both the studies of Repka and Hayward [35, 36] met the criteria for a low risk of bias in the item #7, while in Katsourakis et al. [33] and Karimi and Roshan [34], it was unclear.

Observational studies were assessed with the STROBE checklist. Quasiexperimental studies were considered comparable to prospective cohort studies. All the five studies showed an intermediate quality (Table 2).

### 3.3. Data Extraction

Table 3 presents the principal data of the included studies that analysed the effects of exercise on oxidative stress biomarkers, in people with cancer diagnosis. The geographic origin of the articles was as follows: USA (*n* = 3), Iran (*n* = 1), Ireland (*n* = 1), China (*n* = 1), Greece (*n* = 1), Italy (*n* = 1), Poland (*n* = 1), and Taiwan (*n* = 1). Study characteristics were heterogeneous. Within the included studies, five papers presented an observational design [37–41], and five studies were RCT [32–36]. The sample range varied from 12 to 105 people. Ages ranged from 42-54 to 65–72 years. The length of the intervention varied from 6 weeks to 7 months, the frequency from 2 to 7 times a week. The type of exercise was heterogenous: aerobic training [33, 37, 38], combined exercises [35, 36, 40], endurance training [41], water-based exercises [34], dragon boat racing and walking group [39], and Tai-chi practice [37].

Starting from observational studies results, Jones et al. [37] conducted a quasiexperimental, pilot, single arm study. The population was composed of 16 postsurgical non-small-cell lung cancer patients, with the aim to assess the relationship between an aerobic training of moderate intensity on urinary markers of oxidative status in this specific population. Exercise training comprised three aerobic cycle ergometry weekly sessions for 14 weeks. The intensity increased every week starting from 60% of peak workload in week 1 to 70% in week 14. Interval workouts consisted of 30 s at peak workload followed by 60 s of active recovery for 10–15 intervals. As biomarkers, the investigators assessed F2–isoprostanes, iPF (2 alpha)-III, iPF (2 alpha)-VI, 8,12-iso-iPF (2 alpha)-VI, prostaglandin, 2,3-dinor-iPF (2 alpha)-III, and ametabolite of iPF(2 alpha)-III. An index composed of all the considered F2-isoprostanes isomers increased after the intervention, compared to baseline. Concerning individual isomers, iPF (2-alpha)-III, iPF (2-alpha)-VI, and 8,12-iso-iPF (2 alpha)-VI increased from baseline to postintervention. No change was detected in 2,3-dinoriPF (2 alpha)-III levels.

Tomasello et al. [39] realized a quasiexperimental study, investigating the link between physical exercise on the systemic oxidative status (SOS) in 75 women with breast cancer. The participants were assigned to one of these groups: the control group (resting), dragon boat racing group, and walking group. The walking group consisted in 3-4 hours per week of walking outdoor; the dragon boat racing group attended their session twice a week, for 7 months. All participants followed a supervised fruit/vegetable-rich diet. The investigators assessed oxidant and antioxidant biomarkers, as derivatives of reactive oxygen metabolites (dROMs), determination of lipid hydroperoxides (LPO), biological plasmatic antioxidant potential (BAP) test, total plasmatic thiol groups, SOD activity, and plasmatic glutathione peroxidase (GPx). As secondary outcomes, Tomasello et al. [39] evaluated alkaline and neutral comet assay, human umbilical vein endothelial cell (HUVEC) cultures, isolation of lymphocytes, and DNA repair assay. At the baseline, all women showed high levels of oxidative stress. As major results, exercise kept up the oxidative stress condition, but at the same time, had a positive effect on the antioxidant parameters of the SOS, in particular in the participants who have undergone to the dragon boat racing intervention. DNA fragmentation, according to the levels of single- and double-strand breaks, showed values within the normal range in the participants involved in exercise intervention.

Guinan et al. [38] realized a quasiexperimental, pilot, single arm study, including 12 participants with esophageal cancer. The aim was to verify the impact of a multilevel rehabilitation intervention on inflammation and oxidative stress levels. The intervention was an aerobic training for 12 weeks, 5 times per week. The single session was composed by a warm-up phase, a main aerobic activity, and a cool down phase. Each participant received an individualized dietetic counselling. The assessed biomarkers were as follows: lactate, 8-epimer of prostaglandin F2*α* (8-iso-PGF2*α*), 4-hydroxynonenal (4-HNE); tumour necrosis factor- (TNF-) *α*, interleukin- (IL-) 1*β*, IL-6, IL-8, and 8-hydroxy-deoxyguanosine (8-OHdG). As major findings, IL-8 reduced significantly from baseline to follow-up, and there was a trend towards lower expression patterns of other inflammatory mediators, even if not significant.

Yen et al. [40] aimed to evaluate if exercise could improve physical capacity and reduce oxidative stress, in 30 participants with head and neck cancer treating with chemotherapy. In this noncontrolled study, all participants received a combined exercise intervention for 8 weeks, 3 days per week with 40 to 45 minutes of training time with the following structure: 5 min warm-up, 30 min of aerobic exercise and a 5 min cool down + TheraBand resistance exercise, 10-12 repetitions for set, three sets per training. The intensity was the 60-70% of the maximum heart rate for the aerobic exercise training; between “somewhat heavy” and “heavy” of the Rating Perceived Exertion scale for the resistance exercise. The authors assessed total antioxidant capacity, malondialdehyde (MDA), carbonyl levels, and 8-OHdG levels. As reported, exercise training significantly raised total antioxidant capacity, while plasma concentrations of carbonyl and 8-OHdG diminished after the exercise session. The levels of malondialdehyde did not change.

Finally, Domaszewska et al. [41] realized a pilot study in order to deepen the link between an endurance training intervention and the prooxidative and antioxidant status in 12 women with breast cancer diagnosis, who received a radical mastectomy. The intervention of this single arm study lasted 2 months, 3 times per week, with a 1 hour of training time with a cycle ergometer. Each session was composed by 5 min of warm-up, 30-45 min of the proper part, 5 min of warm-down, and 15 min of stretching and breathing exercises; the adopted intensity was the 50-60% heart rate maximal for the warm-up phase. Exercise loads were individualized based on ergospirometric test. The investigators evaluated the levels of some indicators related to oxidative stress including total phenolics, ferric reducing ability of plasma (FRAP), thiobarbituric acid reactive substances (TBARS), and urea, alongside hematological parameters (erythrocytes, hematocrit, hemoglobin, leukocytes, neutrophils, lymphocytes, monocytes, total proteins, and albumins). This type of intervention did not cause a worsening of oxidative stress in women treated for breast cancer. Analysing included RCT studies, in Karimi and Roshan [34], forty women with breast cancer were assigned into four different groups: the placebo, water-based exercise, ginger supplement, and water-based exercise+ginger supplement groups. The water-based exercise consisted in 10 min warm-up, 20-60 min of water aerobic exercise, and finished with 10 min cool down. This exercise program was scheduled with 4 sessions per week for 6 weeks in total. The evaluated biomarkers were adiponectin, GPx, nitric oxide (NO), and MDA. At the end of the 6 weeks, people who has undergone the water-based exercise showed an improvement of adiponectin, NO and GPx and a reduction in MDA levels.

In Repka and Hayward [35], the investigators aimed to assess the effect of an exercise on muscular strength, cardiorespiratory fitness, and oxidative stress biomarkers in 8 cancer survivors compared with a group of 7 nonexercising cancer patients and a group of 8 age-matched individuals without cancer history. The exercise cancer group attended a 10-week combined exercise program, one hour, 3 days per week. Each session was composed of 5 min of warm-up, 20 min of aerobic exercise, 25 min of resistance training, and 10 min of flexibility and balance training. The intensity was established from 40% to 60% of heart rate reserve and a rating of perceived exertion 4 to 5. No specific diet was prescribed. Trolox-equivalent antioxidant capacity (TEAC), protein carbonyls, and 8-OHdG were assessed. The exercise cancer group showed a significant improvement in antioxidant capacity and a decrease in protein carbonyls at the end of the intervention whereas the nonexercise cancer group did not. No significant within-group changes in 8-OHdG occurred. In 2018, in further investigations within the same study, Repka and Hayward [36] evaluated the effect of their intervention on cancer-related fatigue and the possible relationship with the oxidative stress biomarkers, finding similar results in the same parameters.

Katsourakis et al. [33] focused on evaluating if exercise has any benefit on oxidative stress and glucose levels in 54 patients who undergone a radical pancreatic tumour resection. The intervention group started the training 4 weeks after surgery; this involved 30 min on a bicycle (60% of maximum heart rate) 3 times per week for 12 weeks. The control group did not exercise. The authors assessed uric acid levels, glycosylated haemoglobulin, albumin, and blood glucose pre and post the intervention. The results showed the positive effects of aerobic exercise on glycaemic control, while no change was observed on uric acid, an oxidative stress parameter.

Finally, Jiang et al. [32] evaluated the possible effects of a Tai-Chi intervention on blood oxygen level, and the antioxidant and anti-inflammatory activities, in 100 patients with lung cancer. A simplified 24-posture Yang Tai-Chi was taught by specialized instructors in hospital, i.e., specialized nurses. Tai-Chi was conducted in class, early in the morning, for 60 min divided in 10 min warm-up, 40 min practice, and 10 min cool down) for three months. Jiang et al. assessed serum oxidative parameters, such as total oxidant status (TOS), total antioxidant status (TAS), and the oxidative stress index (OSI); the authors also evaluated some biochemical indexes in serum, such as MDA, SOD, cCAT, and GPx. The results suggest that Tai-Chi exercise improves antioxidant properties in lung cancer patients. After three months, OSI and TOS levels were lower if compared to the control group, while TAS showed higher levels. Tai-Chi also increased the levels of antioxidant markers SOD, CAT, and GPx and reduced the levels of MDA.

## 4. Discussion

Our review systematically analysed ten articles investigating the exercise's effect on oxidative stress biomarkers in adult patients with cancer. In agreement with the evidence outlined in the introduction, three studies [32, 34, 39] have shown the positive effect of exercise on antioxidant enzymes. Karimi and Roshan [34] demonstrated that water-based exercise increased GPx activities in breast cancer patients. Tomasello et al. [39] have also shown that dragon boat racing's exercise significantly raised the levels of GPx and SOD in breast cancer. Jiang et al. [32] observed that Tai-Chi enhanced the blood levels of SOD, CAT, and GPx in lung cancer patients.

It is well known that a rapid and high level of ROS enhances the oxidative damage on DNA that can promote the initiation and progression of cancer [12, 15]. Interestingly, there is evidence that DNA damage promptly arises in white blood cells after an acute endurance exercise training and the DNA damage persist for up 24 h. However, after some postexercise days, the exercise-induced DNA damage is no longer measurable [42]. This biological effect can be due to the effect of exercise in inducing upregulation of DNA repair mechanisms, and it corroborates the concept that exercise causes an adaptive response [6, 42]. 8-OHdG is one of most frequent oxidative DNA lesions that can be observed in various types of cancer [15]. Among the articles meeting the inclusion criteria, only three [36, 38, 40] evaluated the 8-OHdG as biomarker of oxidative stress. Interestingly, Yen et al. [40] found that combined exercise intervention for 8 weeks significantly decreased the 8-OHdG level in people affected by head and neck cancer. In Repka and Hayward's [36] study, a reduction of 8-OHdG level was observed in breast cancer patients after a combined exercise intervention, yet the comparison with the other two study samples is not clear and the results do not allow an exhaustive conclusion. Unfortunately, in the study by Guinan et al. [38], some technical detection problems precluded the fulfilment of the 8-OHdG analysis after exercise. Based on these findings, it is difficult to assess the ability of the exercise intervention to counteract oxidative DNA damage in cancer patients.

To date, different categories of oxidative stress biomarkers are available for assessing the biological effects of exercise in humans [43]. The direct measurement of ROS in the tissue or body fluid is difficult to perform because they are generally too reactive and have a half-life too short [44]. Therefore, to assess the oxidative stress, it is more suitable to investigate the oxidation target products, such as nucleic acid, protein, and lipid or to evaluate the levels of endogenous antioxidants [43]. The most common antioxidant biomarkers include enzymatic antioxidants (CAT, SOD, and GPx), nonenzymatic antioxidants (e.g., GSH and uric acid), and total antioxidant capacity (e.g., TAC) [43]. In this contest, the set of results evaluated in this review gives an encouraging picture of the exercises' benefit in people with cancer. In most studies, the exercise administered to cancer patients improved the indicators of the total antioxidant capacity, enhanced the activity of antioxidant enzymes, or reduced the levels of biomarkers linked to oxidative damage such as MDA and 8-OHdG. Moreover, in our review, the effect of exercise on oxidative stress biomarkers was observed in different types of cancer (lung, breast, head/neck, pancreatic, and oesophageal cancer), and among these, breast and lung cancer are worldwide the most diagnosed [45]. However, the variety of biomarkers found to measure oxidative stress levels (see Table 3) calls for thinking. The heterogeneity of study design hampers the comparison among the articles analysed in this review and limits the ability to draw an exhaustive conclusion on the efficacy of exercise in cancer treatment strategies. This is also in agreement with a previous study indicating that measured oxidative status is variable and sensitive to significant experimental approach differences between research groups [13]. Further research to assess which oxidative stress biomarkers can allow the most valid and reliable measure of change linked to exercise in cancer patients is necessary to broaden outcome integration and to expand the knowledge on this topic of great interest to health care.

Currently, the treatment of cancer is multimodal and includes also PA which is considered a low-cost, safe, and effective strategy [3]. The frequency, intensity, time, and type (FITT) combined to produce total dosage of exercise prescription over a defined period (e.g., weeks or months) and the variation of dosage in the follow-up or within treatment cycles may stimulate greater physiological adaptation also reducing the risk of harm associated to over-training [3]. In the last decades, the scientific community deeply debated the issue if exercise-induced ROS production is a benefit or a disadvantage to health [6]. In connection, a recent study showed that practicing an exercise training regularly does not induce chronic oxidative stress in the involved muscles [23]. Accordingly, different types of exercise in terms of FITT can influence the levels of oxidative stress biomarkers. In the ten studies systematically evaluated, a miscellaneous of exercise interventions were applied in study population affect by different types of cancer. We found differences regarding the FITT of the exercise administered to patients. Combined exercise intervention, including resistance training and aerobic activities, improved the antioxidant status and the profile of the oxidative stress biomarker investigated. The positive influence on oxidative stress level was observed for various types of cancer including breast [34–36, 39, 41], lung [32], pancreatic [33], and head and neck [40]. These findings are promising and support the concept that a combined exercise program, including aerobic and resistance training, should be enclosed in a cancer patient's exercise prescription. Moreover, a combined exercise intervention can well contribute to counter the oxidative damage that arises in the cancer process.

During our investigation about how specific exercise programs can influence the antioxidant status of cancer patients, some issues were identified. One of the limitations of this study is the small number of articles meeting the inclusion criteria and the reduced sample range of the patients enrolled. Additionally, the different study designs (RTC and quasiexperimental study single arm) have restricted the comparison among the selected articles. Finally, the measurement of different biomarkers to evaluate the type of oxidative stress damage or antioxidant capacity of cancer patients has limited the investigators' ability to fully address the study aim.

Despite the limitations observed in the available literature, to our knowledge, this systematic review is the first that have investigated the effects of exercise on oxidative stress biomarkers in various types of cancer. Our findings provide a critical overview of the existing scientific evidence on this topic and point out the need for future studies on this issue.

## 5. Conclusion and Perspectives

Novel approaches need to be evaluated to enhance the prognosis and the quality of life in people with cancer. Our findings indicate that, in the included studies, a miscellaneous of exercise interventions were investigated in terms of FITT of the exercise administered to patients. In this review, we showed that combined exercise intervention with aerobic activities and resistance training can induce positive effects on some oxidative stress biomarkers and enhance the antioxidant status of patients with different oncological diseases such as breast, lung, head and neck, or pancreatic cancer. However, further well-designed high-quality clinical studies focused on different types of cancer are needed to identify a set of suitable biomarkers able to check the physiological impact of the exercise on the antioxidant defences of oncological patients. In connection, the availability of well-established oxidative stress biomarkers is crucial to analyse the efficacy and safety of FITT exercise in thr cancer control approach. Despite our systematic literature search, the findings on the ability of the exercise to counter oxidative DNA damage in cancer patients are limited and preclude a full conclusion. Since the DNA damage has a pivotal role in cancer development and progression, future studies are strongly recommended to evaluate the decrease of genetic damage induced by exercise in cancer patients.

Overall, oxidative stress biomarkers allow a detailed understanding inside the mechanistic effects of exercise benefit in cancer patients. This promising approach can expand the knowledge on the molecular effects on cancer outcomes that different frequencies, intensities, and types of exercise can induce. Admittedly, this information is important to prescribe an appropriate exercise intervention in anticancer strategy. Besides, the identification of tailored and effective exercises based on the diagnosis and prognosis of individual patients will offer new perspective for integrated therapy in oncology.

## Figures and Tables

**Table 1:** PICOST inclusion and exclusion criteria.

Parameter	Inclusion criteria	Exclusion criteria
Population	People with cancer diagnosis	Absence of cancer diagnosis
Intervention	Any type of exercise also combined with pharmacological treatment	Absence of exercise
Comparator	Usual treatment No exercise	
Outcome	Oxidative stress biomarkers levels, physical performance or other indicators of physical fitness	Oxidative stress biomarkers and exercises not assessed
Study design	Experimental or observational study with original primary data	Research studies or other papers with no original data
Timing	English language 10-year publication date limit (May 2011)	Not in English language Published before May 2011

**Table 2:** Quality assessment of RCTs and observational studies.

Authors (year)	Study design	Tool for assessment	Quality
Karimi and Roshan [34] (2012)	RCT	Cochrane ROB tool	Poor
Repka and Hayward [35] (2016)	RCT	Cochrane ROB tool	Poor
Repka and Hayward [36] (2018)	RCT	Cochrane ROB tool	Poor
Katsourakis et al. [33] (2019)	RCT	Cochrane ROB tool	Poor
Jiang et al. [32] (2020)	RCT	Cochrane ROB tool	Good
Jones et al. [37] (2011)	Observational	STROBE	(21/33) intermediate
Guinan et al. [38] (2017)	Observational	STROBE	(18.5/33) intermediate
Tomasello et al. [39] (2015)	Observational	STROBE	(15.5/33) intermediate
Yen et al. [40] (2020)	Observational	STROBE	(18/33) intermediate
Domaszewska et al. [41] (2021)	Observational	STROBE	(16/33) intermediate

**Table 3:** Studies included in the review.

Author, year, country	Study design	Study population	Intervention	Outcomes redox status biomarkers of oxidative stress	Results
Jones et al. [37] 2011 USA	Quasiexperimental pilot study, single arm	*N*: 16 Aged: 64 ± 10 Cancer: Lung	Type: aerobic exercise cycle ergometer session included a 5 min warm-up and 5 min cool down Frequency: 3x week Intensity: 60 to ≥70% of baseline peak workload Time: 14 weeks	Primary outcomes F2–isoprostanes iPF(2 alpha)-III, iPF(2 alpha)-VI, 8,12-iso-iPF(2 alpha)-VI Prostaglandin 2,3-dinor-iPF (2 alpha)-III, ametabolite of iPF (2 alpha)-III Secondary outcomes VO_2_peak Peak workload	iPF(2-alpha)-III Pre: 0.15 ± 0.13 post: 0.24 ± 0.22 Change: +55% *P* = 0, .10 2,3-dinor-iPF (2 alpha)-III Pre: 3.05 ± 2.67, post: 3.63 ± 4.02 Change: +19% *P* = 0.60 iPF (2-alpha)-VI Pre: 2.85 ± 1.33, post: 3.66 ± 2.12 Change: +29% *P* = 0.04 8,12-iso-iPF (2alpha)-VI Pre: 2.12 ± 1.25, post: 2.71 ± 1.84 Change: +28% *P* = 0.07 VO_2_peak Change: 1.13 ± 0.21*P* = 0.14 Peak workload Change: 10 Watts *P* < 0.001

Karimi and Roshan [34] 2012 Iran	RCT	*N*: 40 Aged: 48 ± 6 Cancer: breast CG: placebo EG1: water-base exercise CG: ginger supplementation EG2: Exercises + Ginger supplementation	Type: water-based exercise, 10′ warm up, 20-60′ water aerobic exercise and 10′ cool down Frequency: 4 times per week Time: 6 weeks Type: supplementation 3 capsules 750 mg of ginger rhizome powder Frequency: 4 times per day with breakfast, lunch, dinner and afternoon. Time: 6 weeks	Primary outcomes GPx MDA NO Secondary outcome Adiponectin	GPx CG placebo Pre: 25.1 ± 5.47, post: 24.3 ± 4.62 EG1 exercise Pre: 25.9 ± 2.51, post: 36.9 ± 2.42*P* < 0.05 CG supplementation Pre: 27.3 ± 2.26, post: 31.8 ± 3.61*P* < 0.05 EG2 exercise+supplementation Pre: 26.3 ± 2, post: 44.4 ± 4.79*P* < 0.05 MDA CG placebo Pre: 25.39 ± 5.12, post: 25.05 ± 3.23 EG exercise Pre: 25.55 ± 4.83, post: 21.62 ± 3.2*P* < 0.05 CG supplementation Pre: 24.17 ± 3.68, post: 24.07 ± 3.5 EG2 exercise+supplementation Pre: 23.97 ± 4.02, post: 19.55 ± 3.76*P* < 0.05 NO CG placebo Pre: 36.23 ± 5.96, post: 34.46 ± 4.34 EG1 exercise Pre: 35.26 ± 5.51, post: 41.33 ± 6.22*P* < 0.05 CG supplementation Pre: 35.47 ± 5.55, post: 37.32 ± 4.90 EG2 exercise+supplementation Pre: 36.06 ± 6.27, post: 45.45 ± 7.01*P* < 0.05 Adiponectin CG placebo Pre: 8.43 ± 0.86, post: 7.84 ± 1.03 EG1 exercise Pre: 8.65 + 1, post: 10.45 ± 1.53*P* < 0.05 CG supplementation Pre: 8.03 + 1.0, post: 8.56 ± 1.07 EG2 exercise+supplementation Pre: 8.18 ± 0.74, post: 11.86 ± 0.74

Tomasello et al. [39] 2015 Italy	Quasiexperimental study	*N*: 105 Aged: 51 ± 12 (EG); 49 ± 12 (CGhealthy) Cancer: breast EG-exercises dragon boat: 25 EG-walking: 25 CG: 25 CGhealthy: 30	Type EG-exercise: dragon-boat Type EG-walking: walking Frequency: 3-4 hours 2 per weeks Time: 7 months All patients followed a controlled fruit/vegetable-rich diet	Primary outcomes dROMs BAP LPO SOD activity GPx Total plasmatic thiol groups Secondary outcomes Alkaline and neutral comet assay (% TDNA) Human umbilical vein endothelial cell (HUVEC) cultures, isolation of lymphocytes and DNA repair assay (NER analysis)	dROMS (CARR U) 459 ± 61 CARR U for EG-exercise dragon-boat versus 502 ± 76 CARR U for group EG walking (*P* = 0.332); the increase was significant with respect to the control BrC group for each of the two activity groups *P* = 0.038 and *P* < 0.001, respectively. BAP 2,275 ± 337 *μ*mol/l EG-exercise dragon-boat versus 2,236 ± 223 *μ*mol/l EG walking *P* = ns Following physical training, BAP levels were significantly increased compared with preexercise basal levels and control BrC levels LPO 13.2 ± 3.6 nmol/ml EG-exercise dragon-boat versus 15.08 ± 2.7 nmol/ml EG walking *P* = 0.224 versus 9.7 ± 2.5 nmol/ml control BrC *P* = 0.007 and *P* < 0.001, respectively SOD activity 8.4 ± 1.9 U/ml EG-exercise dragon-boat vs. 6.8 ± 2 U/ml EG-exercise dragon-boat *P* = 0.044 vs. 3.90 ± 2.04 control BrC *P* < 0.001 GPX 246 ± 57.7 nmol/min/ml EG-exercise versus dragon boat, 197 ± 53.3 nmol/min/ml EG-walking versus 147.10 ± 37.6 nmol/min/ml control BrC *P* = 0.007 Total plasmatic thiol groups No differences between EG exercise dragon boat. The control BrC values were also significantly lower than those of the two physical activity groups (both *P* < 0.001). Alkaline and neutral comet assay (%TDNA) EG walking 17.10% versus 14.05% EG exercise dragon boat versus 19.59% control BrC group *P* = ns NER analysis (% TDNA) Dragon boat 31.5 ± 7.6 vs. walking 30.3 ± 8.4% *P* = 0.80 versus 24.5 ± 6% control BrC *P* = 0.008 and *P* = 0.045, respectively, with EG

Repka and Hayward [35] 2016 USA	RCT	*N*: 22 aged: 64.0 ± 10.8 (EX group); 62.4 ± 9 (CG); 55.1 ± 9.7 (CHhealthy) Cancer: different types EG: 8 CG: 7 CGhealthy: 7	Type: combined exercises 5′ warm up 20′ aerobic training 25′ resistance training 10′ flexibility and balance training. Frequency: 3 days per week, 1-hour session. Intensity: 40%-60% of heart rate reserve or a rating of perceived exertion 4 to 5. Time: 10 weeks No diet prescription	Primary outcomes TEAC, Trolox-equivalent antioxidant capacity (mM Trolox) Protein carbonyls (nmol^−1^) 8-OHdG (8-hydroxy-deoxyguanosine (ng ml) Secondary outcomes: composite arm strength (lb) Composite leg strength (lb) Handgrip strength (lb) VO_2_peak (ml)	TEAC EG: Pre: 0.28 ± 0.07*P* < 0.01 than NC Post: 0.39 ± 0.05*P* < 0.01 than baseline CG: Pre: 0.26 ± 0.05*P* < 0.01 than NC Post: 0.32 ± 0.08 CGhealthy 0.37 ± 0.07 Protein carbonyls EG: Pre: 1.30 ± 0.44*P* < 0.05 than NC Post: 0.84 ± 0.33*P* < 0.05 with baseline CG: Pre: 1.18 ± 0.42*P* < 0.05 than NC Post: 1.12 ± 0.22*P* < 0.05 than NC CGhealthy: 0.89 ± 0.25 8-OHdG EG: Pre: 1.47 ± 0.33, post: 0.29 ± 0.18 Significant time by group interaction with control group *P* < 0.05. CG: Pre: 0.35 ± 0.14, post: 0.49 ± 0.22 CGhealthy: 0.33 ± 0.18 Arm strength EX: Pre: 230.4 ± 91.4, post: 324.4 ± 125.2 (significant time by group interaction with control group *P* < 0.05 *P* < 0.01 than baseline CG: Pre: 273.3 ± 148.1, post: 280.1 ± 165.7 Leg strength EG: Pre: 332.3 ± 130.9, post: 445.5 ± 157.2 (significant time by group interaction with control group *P* < 0.05 *P* < 0.01 than baseline CG: Pre: 348.1 ± 166.3, post: 346.9 ± 164.4 Handgrip EX: Pre: 25.8 ± 4.8, post: 28.6 ± 6.0 *P* < 0.05 than baseline CG: Pre: 25.6 ± 9.7, post: 26.8 ± 10.0 VO_2_ EG: Pre: 20.1 ± 9.7, post: 23.4 ± 10.9 *P* < 0.05 than baseline CG: Pre: 18.1 ± 4.4, post: 18.8 ± 6.1

Guinan et al. [38] 2017 Ireland	Quasi-experimental pilot study, single arm	*N*: 12 Aged: 61 ± 7.29 Cancer: oesophageal	Type: aerobic training. Warm up, aerobic exercise and cool down Frequency: 5 times per week Intensity: from 30% to 60% of heart rate reserve Time: 12 weeks Individualised dietetic conselling Group education session	Primary outcomes 4-Hydroxynonenal (4-HNE) 8-Hydroxy-2-deoxyguanosine (8-OHdG) Secondary outcomes Lactate 8-epimer of prostaglandin F2*α* (8-iso-PGF2*α*) Tumour necrosis factor- (TNF-) *α* Interleukin- (IL-) 1*β* IL-6 IL-8 CPET 6MWT MVPA Body weight %BF FM SMM FFM	4-HNE Pre: 1.32 ± 3.96, post: 3.08 ± 5.22*P* < 0.29 8-OHdG Almost all 8-OHdg data was below the assay detection, and therefore, no analysis was completed on this outcome Lactate Pre: 153.61 ± 52.45, post: 182.42 ± 64.99 *P* < 0.23 8-iso-PGF2*α* Pre: 292.95 ± 134.66, post: 259.45 ± 111.19 *P* < 0.20 TNF-*α* Pre: 41.73 ± 309.57, post: 35.92 ± 292.12 *P* < 0.19 IL-1*β* Pre: 3.8 ± 13.93, post: 3.05 ± 12.68*P* < 0.13 IL-6 Pre: 8.69 ± 111.45, post: 6.98 ± 109.93*P* < 0.52 IL-8 Pre: 13.16 ± 88.71, post: 10.14 ± 81.25*P* < 0.03 CPET Pre: 20.08 ± 5.2, post: 24.08 ± 4.99*P* < 0.004 6MWT Pre: 532.17 ± 78.25, post: 588.5 ± 73.14*P* < 0.003 MVPA Pre: 292.91 ± 192.44, post: 317.88 ± 187.05 *P* < 0.363 Body weight Pre: 70.93 ± 19.95, post: 70.28 ± 19.48 *P* < 0.28 %BF Pre: 27.11 ± 5.86, post: 28.22 ± 5.38 *P* < 0.09 FM Pre: 19.48 ± 8.40, post: 19.74 ± 8.04 *P* < 0.57 SMM Pre: 25.44 ± 8.72, post: 24.85 ± 8.97*P* < 0.12 FFM Pre: 50.67 ± 14.99, post: 49.94 ± 15.02*P* < 0.16

Repka and Hayward [36] 2018 USA	RCT	*N*: 22 Aged: 64.0 ± 10.8 (EG); 62.4 ± 9.7 (CG); 55.1 ± 9.7 (CGhealthy) Cancer: different types EG: 8 CG: 7 Healthy HCG: 7	Type: combined exercise, 5′ warm up, 20′ aerobic exercise, 25′ resistance training, and 10′ flexibility and balance training. Frequency: 1 hour session, 3 days per week Intensity: from 40% to 60% of heart rate reserve or a rating of perceived exertion 4 to 5. Time:10 weeks No diet prescription	Primary outcomes TEAC, Trolox-equivalent antioxidant Protein carbonyls 8-OHdG (8-hydroxy-deoxyguanosine) Secondary outcome Piper Fatigue Inventory	TEAC EG: Pre: 0.75 ± 0.19, post: 1.06 ± 0.13 *P* < 0.05 with baseline CG: Pre: 0.69 ± 0.16, post: 0.85 ± 0.22 CGhealthy 1.0 ± 0.20*P* < 0.5 with cancer Protein carbonyls: EG: Pre: 1.46 ± 0.49, post: 0.94 ± 0.37 *P* < 0.05 with baseline CG: Pre: 1.32 ± 0.48, post: 1.26 ± 0.25 CGhealthy: 1.0 ± 0.28*P* < 0.5 with cancer 8-OHdG EG: Pre: 1.45 ± 0.96, post: 0.88 ± 0.55 significant time by group interaction with control group *P* < .05. CG: Pre: 1.08 ± 0.44, post: 1.48 ± 0.66 CGhealthy: 1.0 ± 0.57 CRF: EG: Pre: 5.0 ± 2.2, post: 2.6 ± 1.9*P* < 0.05 with baseline CG: Pre: 4.7 ± 2.5, post: 3.2 ± 2.4 CGhealthy 1.0 ± 1.0*P* < 0.5 with cancer

Katsourakis et al. [33] 2019 Greece	RCT	*N*: 54 Aged 59.90 EG (range 54.67-65.14) 69.14 CG (range: 65.73–72.55) Cancer: pancreas EG: 28 CG: 26	Type: aerobic exercise Intensity: 60% of maximum heart rate) Frequency: 30 min, 3 per week Time: 12 weeks.	Primary outcomes Uric acid levels Secondary outcomes Haemoglobulin Abumin Blood glucose Glycosylated	Uric acid EG vs. CG *P* = 0.069 > 0.05 The statistics illustrated that exercise can have a positive influence on glycaemic control, but no influence was observed on the levels of uric acid, which represents oxidative stress. Glucose EG vs. CG *P* < 0.05

Jiang et al. [32] 2020 China	RCT	*N*: 100 Aged: 59.30 ± 7.40 (EG); 57.56 ± 11.23 (CG) EG: 50 CG: 50 Cancer: lung	Type: Tai-Chi 10-min warm-up 40-min practice 10 min cool down Frequency: 60 min, 92 lessons Time: 3 months	Primary outcomes TOS TAS OSI MDA, SOD, CAT, GPx TNF-*α*, IL-1, and IL-6 Secondary outcome VAS PHS	Pre: no statistical differences for TOS TAS and OSI between the two groups *P* > 0.05. Post: the levels for TOS and OSI in the EG group were lower than those in the CG while TAS level in the EG was higher than in the CG *P* < 0.05. EG vs. CG pre: no statistical differences For SOD, CAT, GSPx and MDA *P* > 0.05. EG vs. CG post SOD, CAT, and GSPx increased while the serum level of MDA was reduced in the EG vs. CG *P* < 0.05 EG vs. CG pre: no statistical differences for TNF-*α*, IL-1, IL-6 and IL-10 *P* > 0.05. EG vs. CG post TNF-*α*, IL-1, and IL-6 were reduced while the serum level of IL-10 was increased in the EG vs. CG *P* < 0.05. VAS EG: Pre: 75.86 ± 7.36, post: 50.96 ± 8.23 CG: Pre: 82.46 ± 6.52, post: 78.01 ± 7.9*P* < 0.001 PHS EG: Pre: 3.15 ± 0.62, post: 2.03 ± 0.77 CG: Pre: 2.93 ± 0.54, post: 2.68 ± 0.69*P* < 0.003

Yen et al. [40] 2020 Taiwan	Quasiexperimental study, single arm	*N*: 42 Aged: 56.0 ± 12.3 Cancer: head/neck	Type: 5 min warm up, 30 min of aerobic exercise training and a 5 min cool down + TheraBand resistance exercise, 10 to 12 repetitions for one set, three sets per training, both upper and lower extremities. Frequency: 3 days per week. 40 to 45 minutes of training time. Time: 8 weeks Intensity: 60-70% of the maximum heart rate	Primary outcomes Total antioxidant capacity Malondialdehyde Carbonyl levels 8-Hydroxy-20-deoxyguanosine (8-OHdG)	Total antioxidant capacity Pre: 221.7 ± 62.2, post: 443.7 ± 72.1 Malondialdehyde Pre: 4.7 ± 0.8, post: 3.8 ± 1.3 Carbonyl levels Pre: 10.1 ± 2.6, post: 5.5 ± 1.8 8-Hydroxy-20-deoxyguanosine (8-OHdG) Pre: 1031.3 ± 43.8, post: 761.3 ± 66.3

Domaszewska et al. [41] 2021 Poland	Quasiexperimental pilot study, single arm	N: 12, Aged: 50.6 ± 2.9 Cancer: breast	Type: Endurance training 5 min of warm up, 30-45 min of the proper part, 5 min of warm-down, 15 min of stretching and breathing exercises Frequency: 3 times per week. 1 hour of training time with a cycle ergometer. Time: 8 weeks Intensity: 50-60% HRmax for the warm-up phase. Exercise loads were determined individually on the basis of ergospirometric exercise test	Primary outcomes Total phenolics FRAP TBARS Urea Secondary outcomes VT heart rate VT load Peak HR Peak VO_2_ Peak load	Total phenolics Pre: 2.44 ± 0.09, post: 2.43 ± 0.28 FRAP Pre: 857.25 ± 147.17, post: 859.67 ± 148.65 TBARS Pre: 5.09 ± 2.09, post: 5.02 ± 1.81 Urea Pre: 3.32 ± 2.09, post: 3.71 ± 1.99 VT heart rate Pre: 127.75 ± 13.07, post: 142.25 ± 13.06 VT load Pre: 76.67 ± 13.37, post: 94.17 ± 14.29 Peak HR Pre: 158.92 ± 15.37, post: 166.50 ± 13.56 Peak VO_2_ Pre: 25.74 ± 4.04, post: 27.00 ± 3.68 Peak load Pre: 112.50 ± 23.01, post: 123.33 ± 22.09

Hydroperoxides; SOD: superoxide dismutase; TEAC: Trolox-equivalent antioxidant capacity; 8-OHdG: 8-hydroxy-deoxyguanosine; 8-iso-PGF2*α*: 8-epimer of prostaglandin F2*α*; 4-HNE: 4-hydroxynonenal; TNF-*α*: tumour necrosis factor-*α*; IL-1*β*: interleukin-1*β*; IL-6: interleukin-6; IL-8: interleukin-8; CPET: cardiopulmonary exercise testing; 6MWT: six-minute walk test; MVPA: moderate to vigorous physical activity; %BF: % body fat; SMM: skeletal muscle mass; FFM: fat-free mass; TOS: total oxidant status; TAS: total antioxidant status; OSI: oxidative stress index; VAS: visual analogic scale; PHS: Prince-Henry score method; FRAP: ferric reducing ability of plasma; TBARS: thiobarbituric acid reactive substances; VT: ventilatory threshold; HR: heart rate.

## Data Availability

No data were used to support this study.

## References

[1] Campbell K. L., Winters-Stone K. M., Wiskemann J. (2019). Exercise guidelines for cancer survivors: consensus statement from international multidisciplinary roundtable. *Medicine & Science in Sports & Exercise*.

[2] Caspersen C. J., Powell K. E., Christenson G. M. (1985). Physical activity, exercise, and physical fitness: definitions and distinctions for health-related research. *Public Health Reports*.

[3] Hayes S. C., Newton R. U., Spence R. R., Galvão D. A. (2019). The Exercise and Sports Science Australia position statement: exercise medicine in cancer management. *Journal of Science and Medicine in Sport*.

[4] Fuller J. T., Hartland M. C., Maloney L. T., Davison K. (2018). Therapeutic effects of aerobic and resistance exercises for cancer survivors: a systematic review of meta-analyses of clinical trials. *British Journal of Sports Medicine*.

[5] Friedenreich C. M., Neilson H. K., Farris M. S., Courneya K. S. (2016). Physical activity and cancer outcomes: a precision medicine approach. *Clinical Cancer Research*.

[6] Powers S. K., Deminice R., Ozdemir M., Yoshihara T., Bomkamp M. P., Hyatt H. (2020). Exercise-induced oxidative stress: friend or foe?. *Journal of Sport and Health Science*.

[7] Johnsson A., Broberg P., Krüger U., Johnsson A., Tornberg Å. B., Olsson H. (2019). Physical activity and survival following breast cancer. *European Journal of Cancer Care*.

[8] Nascimento W., Ferrari G., Martins C. B. (2021). Muscle-strengthening activities and cancer incidence and mortality: a systematic review and meta-analysis of observational studies. *International Journal of Behavioral Nutrition and Physical Activity*.

[9] Hojman P., Gehl J., Christensen J. F., Pedersen B. K. (2018). Molecular mechanisms linking exercise to cancer prevention and treatment. *Cell Metabolism*.

[10] Sasso J. P., Eves N. D., Christensen J. F., Koelwyn G. J., Scott J., Jones L. W. (2015). A framework for prescription in exercise-oncology research. *Journal of Cachexia, Sarcopenia and Muscle*.

[11] Ballard-Barbash R., Friedenreich C. M., Courneya K. S., Siddiqi S. M., McTiernan A., Alfano C. M. (2012). Physical activity, biomarkers, and disease outcomes in cancer survivors: a systematic review. *Journal of the National Cancer Institute*.

[12] Thomas R. J., Kenfield S. A., Jimenez A. (2017). Exercise-induced biochemical changes and their potential influence on cancer: a scientific review. *British Journal of Sports Medicine*.

[13] Arena S. K., Doherty D. J., Bellford A., Hayman G. (2019). Effects of aerobic exercise on oxidative stress in patients diagnosed with cancer: a narrative review. *Cureus*.

[14] Sosa V., Moliné T., Somoza R., Paciucci R., Kondoh H., Lleonart M. E. (2013). Oxidative stress and cancer: an overview. *Ageing Research Reviews*.

[15] Arfin S., Jha N. K., Jha S. K. (2021). Oxidative stress in cancer cell metabolism. *Antioxidants (Basel)*.

[16] Assi M., Dufresne S., Rébillard A. (2020). Exercise shapes redox signaling in cancer. *Redox Biology*.

[17] Thirupathi A., Wang M., Lin J. K. (2021). Effect of different exercise modalities on oxidative stress: a systematic review. *BioMed Research International*.

[18] Musci R. V., Hamilton K. L., Linden M. A. (2019). Exercise-induced mitohormesis for the maintenance of skeletal muscle and healthspan extension. *Sports*.

[19] Bouviere J., Fortunato R. S., Dupuy C., Werneck-de-Castro J. P., Carvalho D. P., Louzada R. A. (2021). Exercise-stimulated ROS sensitive signaling pathways in skeletal muscle. *Antioxidants*.

[20] Hayes J. D., Dinkova-Kostova A. T. (2014). The Nrf2 regulatory network provides an interface between redox and intermediary metabolism. *Trends in Biochemical Sciences*.

[21] Suzuki T., Motohashi H., Yamamoto M. (2013). Toward clinical application of the Keap1-Nrf2 pathway. *Trends in Pharmacological Sciences*.

[22] Smolková K., Mikó E., Kovács T., Leguina-Ruzzi A., Sipos A., Bai P. (2020). Nuclear factor erythroid 2-related factor 2 in regulating cancer metabolism. *Antioxidants & Redox Signaling*.

[23] Theofilidis G., Bogdanis G. C., Koutedakis Y., Karatzaferi C. (2018). Monitoring exercise-induced muscle fatigue and adaptations: making sense of popular or emerging indices and biomarkers. *Sports*.

[24] Moher D, Liberati A, Tetzlaff J, Altman DG, Group P (2009). Preferred reporting items for systematic reviews and meta-analyses: the PRISMA statement. *BMJ*.

[25] Higgins J. G., Green S. (2008). *Cochrane Handbook for Systematic Reviews of Interventions.*.

[26] Greenhalgh T., Peacock R. (2005). Effectiveness and efficiency of search methods in systematic reviews of complex evidence: audit of primary sources. *BMJ*.

[27] Sterne J. A. C., Savović J., Page M. J. (2019). RoB 2: a revised tool for assessing risk of bias in randomised trials. *BMJ*.

[28] Cuschieri S. (2019). The STROBE guidelines. *Saudi Journal of Anaesthesia*.

[29] Salehi N., Janjani P., Tadbiri H., Rozbahani M., Jalilian M. (2021). Effect of cigarette smoking on coronary arteries and pattern and severity of coronary artery disease: a review. *The Journal of International Medical Research*.

[30] Sahebi A., Jahangiri K., Sohrabizadeh S., Golitaleb M. (2019). Prevalence of workplace violence types against personnel of emergency medical services in Iran: a systematic review and meta-analysis. *Iranian Journal of Psychiatry*.

[31] Biagi C., Nunzio M. D., Bordoni A., Gori D., Lanari M. (2019). Effect of adherence to Mediterranean diet during pregnancy on children's health: a systematic review. *Nutrients*.

[32] Jiang M., Zhao H., Liu J., Zhao X., Jin L., Pan R. (2020). Does Tai Chi improve antioxidant and anti-inflammatory abilities via the KEAP1-NRF2 pathway and increase blood oxygen level in lung cancer patients: a randomized controlled trial?. *European Journal of Integrative Medicine*.

[33] Katsourakis A., Vrabas I., Dimitriadis C. (2019). How exercise can influence oxidative stress and glucose levels after pancreatic resection: a randomised controlled trial. *Digestive Surgery*.

[34] Karimi N., Roshan V. D. (2013). Change in adiponectin and oxidative stress after modifiable lifestyle interventions in breast cancer cases. *Asian Pacific Journal of Cancer Prevention*.

[35] Repka C. P., Hayward R. (2016). Oxidative stress and fitness changes in cancer patients after exercise training. *Medicine & Science in Sports & Exercise*.

[36] Repka C. P., Hayward R. (2018). Effects of an exercise intervention on cancer-related fatigue and its relationship to markers of oxidative stress. *Integrative Cancer Therapies*.

[37] Jones L. W., Eves N. D., Spasojevic I., Wang F., Il'yasova D. (2011). Effects of aerobic training on oxidative status in postsurgical non-small cell lung cancer patients: a pilot study. *Lung Cancer*.

[38] Guinan E. M., Doyle S. L., O’Neill L. (2016). Effects of a multimodal rehabilitation programme on inflammation and oxidative stress in oesophageal cancer survivors: the ReStOre feasibility study. *Supportive Care in Cancer*.

[39] Tomasello B., Malfa G. A., Strazzanti A. (2017). Effects of physical activity on systemic oxidative/DNA status in breast cancer survivors. *Oncology Letters*.

[40] Yen C.-J., Hung C.-H., Tsai W.-M. (2020). Effect of exercise training on exercise tolerance and level of oxidative stress for head and neck cancer patients following chemotherapy. *Frontiers in Oncology*.

[41] Domaszewska K., Janiak A., Podgórski T. (2021). A pilot study of influence of endurance training on the prooxidative and antioxidant status of women after breast cancer. *International Journal of Environmental Research and Public Health*.

[42] Tryfidou D. V., McClean C., Nikolaidis M. G., Davison G. W. (2020). DNA damage following acute aerobic exercise: a systematic review and meta-analysis. *Sports Medicine*.

[43] Gomez-Cabrera M. C., Carretero A., Millan-Domingo F. (2021). Redox-related biomarkers in physical exercise. *Redox Biology*.

[44] Dalle-Donne I., Rossi R., Colombo R., Giustarini D., Milzani A. (2006). Biomarkers of oxidative damage in human disease. *Clinical Chemistry*.

[45] Sung H., Ferlay J., Siegel R. L. (2021). Global cancer statistics 2020: GLOBOCAN estimates of incidence and mortality worldwide for 36 cancers in 185 countries. *CA: a Cancer Journal for Clinicians*.

